# Role of Dapagliflozin and Liraglutide on Diabetes-Induced Cardiomyopathy in Rats: Implication of Oxidative Stress, Inflammation, and Apoptosis

**DOI:** 10.3389/fendo.2022.862394

**Published:** 2022-03-18

**Authors:** Mohamed El-Shafey, Mosaab Salah El-din El-Agawy, Mohamed Eldosoky, Hasnaa Ali Ebrahim, Dalia Mahmoud Abdelmonem Elsherbini, Mohamed El-Sherbiny, Saad Mohamed Asseri, Nehal M. Elsherbiny

**Affiliations:** ^1^ Department of Anatomy and Embryology, Faculty of Medicine, Mansoura University, Mansoura, Egypt; ^2^ Physiological Sciences Department, Fakeeh College for Medical Sciences, Jeddah, Saudi Arabia; ^3^ Department of Neuroscience Technology-College of Applied Sciences, Jubail Imam Abdulraman bin Faisal University, Dammam, Saudi Arabia; ^4^ Department of Basic Medical Sciences, College of Medicine, Princess Nourah bint Abdulrahman University, Riyadh, Saudi Arabia; ^5^ Department of Clinical Laboratory Sciences, College of Applied Medical Sciences, Jouf University, Sakaka, Saudi Arabia; ^6^ Department of Basic Medical Sciences, College of Medicine, AlMaarefa University, Riyadh, Saudi Arabia; ^7^ Department of Clinical Medical Sciences, College of Medicine, AlMaarefa University, Riyadh, Saudi Arabia; ^8^ Department of Biochemistry, Faculty of Pharmacy, Mansoura University, Mansoura, Egypt; ^9^ Department of Pharmaceutical Chemistry, Faculty of Pharmacy, University of Tabuk, Tabuk, Saudi Arabia

**Keywords:** dapagliflozin (PubChem CID: 9887712), liraglutide (PubChem CID: 16134956), diabetes risk, cardiomyopathy, rats

## Abstract

The current study aims to assess the protective effects of dapagliflozin (Dapa; a sodium-glucose cotransporter-2 inhibitor) and/or liraglutide (Lira; a glucagon-like peptide 1 agonist) in an experimental model of diabetic cardiomyopathy (DCM). A single dose of streptozotocin (STZ) was administrated to male Sprague–Dawley rats by intraperitoneal injection at a dose of 50 mg/kg to induce diabetes mellitus (DM). Dapa (1 mg/kg, orally), Lira (0.4 mg/kg, s.c.), and Dapa–Lira combination were administrated for 8 weeks once-daily. Blood samples were evaluated for glucose level and biochemical markers of cardiac functions. Cardiac tissue was dissected and assessed for redox homeostasis (malondialdehyde (MDA), glutathione (GSH), and catalase (CAT)), pro-inflammatory mediators (NF-κB and tumor necrosis factor-α (TNF-α)), and apoptotic effectors (caspase-3). Moreover, the effect of treatments on the cardiac cellular structure was studied. Dapa and/or Lira administration resulted in significant improvement of biochemical indices of cardiac function. Additionally, all treatment groups demonstrated restoration of oxidant/antioxidant balance. Moreover, inflammation and apoptosis key elements were markedly downregulated in cardiac tissue. Also, histological studies demonstrated attenuation of diabetes-induced cardiac tissue injury. Interestingly, Dapa–Lira combination treatment produced a more favorable protective effect as compared to a single treatment. These data demonstrated that Dapa, Lira, and their combination therapy could be useful in protection against DM-accompanied cardiac tissue injury, shedding the light on their possible utilization as adjuvant therapy for the management of DM patients.

## Introduction

Diabetes mellitus (DM) is a complex chronic metabolic disease whose incidence is escalating globally (1, 2). Type 1 DM (T1DM) is caused by progressive T cell-mediated immune damage of pancreatic β cells resulting in persistent hyperglycemia (3). On the other hand, type 2 DM (T2DM) is associated with deficient insulin secretion due to compromised β-cell function accompanied by peripheral insulin secretion. However, recent reports proposed β-cell as a key contributor to the pathogenesis of T1DM *via* evading the immune attack, highlighting the contribution of β-cell stress responses to disease onset (4). Thereby, several therapeutic interventions that aimed at improving glycemic control and ameliorating pressure exerted on β cells in T2DM have been evaluated in the context of T1DM (5). Among these strategies, glucagon-like peptide-1 (GLP-1) analogs and sodium-glucose cotransporter-2 (SGLT2) inhibitors have recently shown some benefit as adjuvant therapy with insulin in the treatment of patients with T1DM (6). Further, intensive insulin therapy in T1DM increases the occurrence of abdominal obesity, dyslipidemia, and hypertension, putting them at higher risk of cardiovascular disorders. Hence, antihyperglycemic classes including GLP-1 analogs and SGLT2 inhibitors hold promise as additional adjunctive therapy options that complement insulin efficacy, reduce the risk of weight gain, and improve overall glycemic control (7).

Dapagliflozin (Dapa) is an SGLT2 inhibitor that prevents renal glucose reabsorption in proximal tubules. Thus, it reduces the blood glucose level only when it exceeds a reduced renal threshold, decreasing the incidence of hypoglycemia. Additionally, urinary loss of glucose helps weight reduction (8). Liraglutide (Lira) is a synthetic long-acting GLP-1 receptor agonist that has a high structural similarity to human GLP-1. It acts by reducing glucagon secretion, suppressing appetite, slowing gastric emptying, and helping weight loss (9).

Several clinical trials were conducted to assess the utility of using SGLT2 inhibitors and GLP-1 receptor agonists as adjuncts to insulin therapy for T1DM patients. However, the results were not conclusive. This can be explained by the small sample size and the unsatisfactory glucose-lowering efficacy of these agents (10). Dapa has now been licensed for clinical use as adjuvant therapy to insulin in Europe and Japan. However, it has not been approved in the United States due to the increased risk of diabetic ketoacidosis (DKA) (11). However, a risk mitigation strategy has been developed for reducing DKA in T1DM patients treated with SGLT2 inhibitors (12). On the other hand, the use of Lira in combination with insulin resulted in a smaller HbA1c decrease; therefore, it was not considered for a license. Nevertheless, using various combinations of inhibitors of SGLT2 and agonists of GLP-1 receptor is currently under investigation to test whether this approach would yield better therapeutic outcomes in T1DM patients. In this context, a clinical trial conducted by Kuhadiya et al. demonstrated significant improvement in glycemic control and body weight when Dapa was added to Lira and insulin for the treatment of T1DM patients (13). However, two patients developed DKA. With the reported suppressive effect Lira on ketogenesis (14), using a lower dose of SGLT2 inhibitors in combination can achieve the needed balance between clinical benefit and increased risk of DKA. Indeed, the combined beneficial effects of SGLT2 inhibitors and GLP-1 receptor agonists on metabolic indices and vascular complications need further investigations for further establishment of this combination in the treatment of T1DM patients.

Uncontrolled DM is accompanied by a lot of complications that affect various body organs. Among diabetes-associated organ complications, diabetic cardiomyopathy (DCM) is a major leading cause of death in diabetic patients (15). It is characterized by diastolic and systolic dysfunction and pathological cardiac remodeling that may end in heart failure. Various preclinical studies delineated multiple intracellular pathways that are implicated in the pathogenesis of DCM including endoplasmic reticulum stress, oxidative stress, impaired calcium handling, increased lipid utilization, and activation of inflammatory pathways (16). The use of SGLT2 inhibitors and GLP-1 receptor agonists in T2DM has improved associated disorders in various preclinical (17), and clinical studies (18). However, the effect of these antidiabetic drugs on organ injury in T1DM needs further investigation. The present study aimed to study the protective effects of Dapa and/or Lira in an experimental model of DCM. Further, the potential underlying molecular mechanisms have been evaluated.

## Materials and Methods

### Experimental Design and Treatments

All used protocols were approved by the guidelines of the Ethics Committee at the Faculty of Medicine, Mansoura University, Mansoura, Egypt. Fifty male Sprague–Dawley (SD) rats were obtained from the laboratory animal unit of the Urology and Nephrology Center, Mansoura, Egypt, and kept under required conditions of temperature (21°C ± 2°C), humidity (50% ± 10%), and light (12 h light/dark cycle) with *ad libitum* access to distilled water and a standard rat diet. After 1-week acclimatization, a single intraperitoneal injection of streptozotocin (STZ) at a dose of 50 mg/kg was used to induce T1DM as previously described (19). Hyperglycemia incidence was confirmed 3 days later *via* assessment of blood glucose level using a glucometer (Accu-Check, Roche, Mannheim, Germany). Rats were considered diabetic when blood glucose level was greater than 250 mg/dl. The diabetic rats were further randomly assigned into subgroups (n = 10 for each group), including a saline group, a Dapa group (FORXIGA, AstraZeneca, Mississauga, ON, Canada, 1 mg/kg/day, orally), a Lira group (VICTOZA, Novo Nordisk, Bagsværd, Denmark, 0.4 mg/kg/day, s.c.), and a Dapa+Lira group. Each rat was given treatment for 8 weeks. An additional group of rats (n = 10) was used as the normal control group and received saline.

At the end of the experimental period, blood glucose level was assessed as previously mentioned. Animals were sacrificed under anesthesia, and blood samples were withdrawn from the retro-orbital plexus and centrifuged at 3,000 rpm for 10 min to separate the serum, which was further used for biochemical analysis. The hearts were rapidly isolated. One part was fixed in 10% formalin for histopathological examination and immunohistochemistry (IHC) analysis. Another part was washed with ice-cold saline, rapidly frozen in liquid nitrogen, and stored at −80°C for protein and RT-PCR assays.

### Biochemical Measurements

The serum biochemical profiles, including insulin (Cloud-Clone Corp., Houston, TX, USA), creatine kinase-MB (CK-MB) (bioMérieux Diagnostics, Milan, Italy), lactate dehydrogenase (LDH; Spectrum Diagnostic Company, Cairo, Egypt) were evaluated according to the manufacturers’ instructions.

### Histological Studies

Dissected organ specimens were fixed and dehydrated in ascending grades of ethanol. Thereafter, cardiac specimens were embedded in paraffin wax. Then, 5-µm-thick sections were prepared from paraffin blocks. Sections were further stained with H&E to be assessed for histopathological alterations. The examination was performed by a qualified observer without the identification of the experimental groups. All records were performed using Olympus light microscope equipped with a digital camera (Tokyo, Japan). For morphometric analysis, semiquantification of myocardial injury was performed (20, 21),. Each slide was inspected for cardiac pathological changes in three high-power fields using the following scoring system for grading the cardiomyopathy severity: 0 = no damage, 1 = mild lesion, 2 = moderate lesion, and 3 = severe lesion, with (1+) for the presence of myocardial fiber swelling and interstitial edema, (1+) for disorganization of myocardial fiber with or without fibroblastic proliferation, (1+) for perinuclear vacuolization or myocardial fiber vacuolization, (1+) for myocardial fibers myocytolysis/necrosis, and 0 when there was no damage noted.

### Preparation of Cardiac Tissue Homogenate and Assessment of Oxidative Stress

Cardiac tissue was homogenized in phosphate-buffered saline (PBS) to prepare 10% (w/v) homogenate using Omni-125 handheld homogenizer (Omni International, Kennesaw, GA, USA). The homogenates were further spun at 5,000*g* for 15 min at 4°C. Oxidative stress biomarkers were then assessed in freshly prepared supernatants. Levels of malondialdehyde (MDA), a marker of lipid peroxidation, and the antioxidant reduced glutathione (GSH) in addition to the activity of the antioxidant enzyme catalase (CAT) were measured in prepared tissue homogenates assayed using commercially available kits by Bio Diagnostic (Giza, Egypt) according to the manufacturer’s protocols.

### Assessment of Cardiac Inflammatory Cytokine Levels by ELISA

Levels of interleukin-1β (IL-β) and interleukin-6 (IL-6) in cardiac tissue homogenates prepared from different experimental groups were assessed by the ELISA method according to the manufacturer’s instructions (Cloud-Clone Corp., Houston, TX, USA).

### Real-Time PCR

Total RNA was extracted from cardiac tissues from all experimental groups using Direct-zol RNA Miniprep Plus (ZYMO RESEARCH CORP., Irvine, CA, USA, Cat# R2072). Extracted RNA was assessed for quantity and quality by spectrophotometry using Beckman dual spectrophotometer (Brea, CA, USA).

Extracted RNA was then utilized for reverse transcription into complementary DNA (cDNA) using the SuperScript IV One-Step RT-PCR kit (Thermo Fisher Scientific, Waltham, MA, USA, Cat# 12594100). cDNA amplification was performed using a 48-well plate StepOne instrument (Applied Biosystems, Foster City, CA, USA). The thermal profile included the following: reverse transcription for 10 min at 45°C, RT inactivation and initial denaturation by 40 cycles of 10 s at 98°C, and an amplification step for 10 s at 55°C followed by 30 s at 72°C. Data were then expressed in cycle threshold (Ct) for the housekeeping gene and the target genes. ΔΔCt method was used to normalize variation in the target genes expression by referring to the expression value of mean critical threshold (CT) of a housekeeping gene. Primer sequence for tumor necrosis factor-α (TNF-α) gene was forward 5′-TAC TGA ACT TCG GGG TGA TTG GTC C-3′ and reverse 5′-CAG CCT TCT CCC TTG AAG AGA ACC-3′, for caspase-3 gene was forward 5′-ATGGACAACAACGAAACCTC-3′ and reverse 5′-TTAGTGATAAAAGTACAGTTCTT-3, and for β-actin housekeeping gene was forward 5′-CTAAGGCCAACCGTGAAAAG-3′ and reverse 5′-GCCTGGATGGCTACGTACA-3′. The relative quantitation (RQ) of each target gene is performed based on the calculation of the 2^−ΔΔCt^ method.

### Immunohistochemistry

Sections were dewaxed, rehydrated, and washed, followed by incubation in 3% hydrogen peroxide (H_2_O_2_, 3%) for 10 min and then blocking with 5% bovine serum albumin (BSA) in Tris-buffered saline (TBS). Thereafter, immunolocalization was performed by incubation with antibodies for NF-κB/p56 (Thermo Fisher Scientific Inc., Waltham, MA, USA), TNF-α (sc-52746, Santa Cruz, Paso Robles, CA, USA), and cleaved caspase-3 (GB11532, Wuhan Servicebio Biotechnology, Wuhan, China) at 4°C overnight. Then, sections were washed in TBS 3 times, followed by incubation with secondary antibodies. After washing in TBS, diaminobenzidine/peroxidase was used for development and hematoxylin for counter-staining. The sections were then mounted and examined using Olympus light microscope equipped with a digital camera (Tokyo, Japan). For IHC quantitative assessment, an immunoreactive score (IRS) was used. It provides a scale of 0–12 representing IRS index (0–1 = negative, 2–3 = mild, 4–8 = moderate, and 9–12 = strongly positive). IRS is obtained by multiplication between staining intensity grading (0–3) and positive cells proportion grading (0–4) (22) and quantified using the QuPath program (0.1.2) (23).

### Statistical Analysis

Analysis and graphical representation of data were accomplished by GraphPad prism statistical software (version 8, USA). Results are expressed as mean ± SE, and statistical analysis was performed using one-way ANOVA followed by a *post-hoc* test (Tukey–Kramer). Two-way ANOVA was used to calculate statistical significance for NF-κB/p56 nuclear and cytoplasmic expression among various experimental groups. Statistical significance was considered at *p* < 0.05.

## Results

### Effects of Dapagliflozin–Liraglutide Treatment on Blood Glucose and Serum Insulin Levels

**Figure 1:**
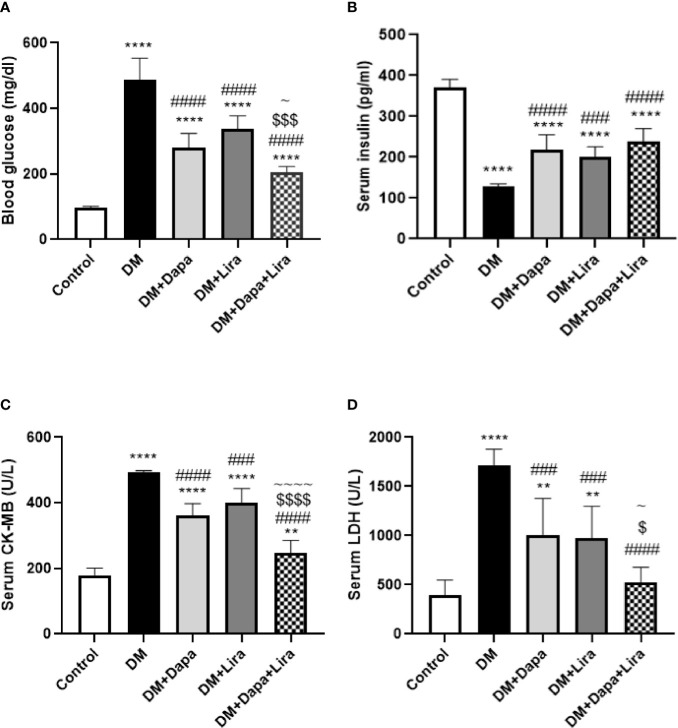
Effect of dapagliflozin (Dapa) and liraglutide (Lira) or their combination on **(A)** blood glucose level, **(B)** serum creatine kinase-MB (CK-MB), and **(C)** serum lactate dehydrogenase (LDH). Data are represented as mean ± SE, n = 6. ** significance in comparison with control group at *p* < 0.01, **** at *p* < 0.0001, ### significance in comparison with diabetic group at *p* < 0.001, #### at *p* < 0.0001, ~ significance in comparison with DM+Dapa at *p* < 0.05, ~~~~ significance in comparison with DM+Dapa at *p* < 0.0001, $ significance in comparison with DM+Lira group at *p* < 0.05, $$$ at *p* < 0.001, $$$$ at *p* < 0.0001.

Induction of experimental DM resulted in a 4.99-fold (*p* < 0.0001) increase in blood glucose level accompanied by a 65.3% reduction (*p* < 0.0001) in serum insulin as compared to normal control. Treatment with Dapa and Lira for 8 weeks resulted in 42.5% and 30.9% significant decrease (*p* < 0.0001) in blood glucose level and 1.69- and 1.55-fold (*p* < 0.0001, *p* < 0.001) increase in serum insulin level, respectively, as compared to the diabetic group. Combined treatment with Dapa and Lira significantly improved blood glucose level (*p* < 0.05, *p* < 0.0001, respectively) and produced non-significant elevation in serum insulin level as compared to single treatment groups (**Figures 1A, B**).

### Effects of Dapagliflozin–Liraglutide Treatment on Biomarkers of Cardiac Injury

Serum levels of LDH and CK-MB were significantly increased by 2.47- and 4.31-fold (*p* < 0.0001 and *p* < 0.0001), respectively, in the DM group compared to the normal group. However, these levels were significantly reduced by 26.5% and 41.2% (*p* < 0.0001, *p* < 0.001), respectively, in the Dapa-treated group and by 18.7% and 43.2% (*p* < 0.001), respectively, in the Lira-treated group when compared to the diabetic group. Combined Dapa–Lira therapy produced more reduction in the levels of these cardiac markers by 31.6% and 47.7% (*p* < 0.0001, *p* < 0.05, respectively), as compared to the DM+Dapa group and by 38% and 45.9% (*p* < 0.0001, *p* < 0.05, respectively) as compared to the DM+Lira group (**Figures 1C, D**).

### Effects of Dapagliflozin–Liraglutide Treatment on Type 1 Diabetes Mellitus-Induced Cardiac Histological Changes

**Figure 2:**
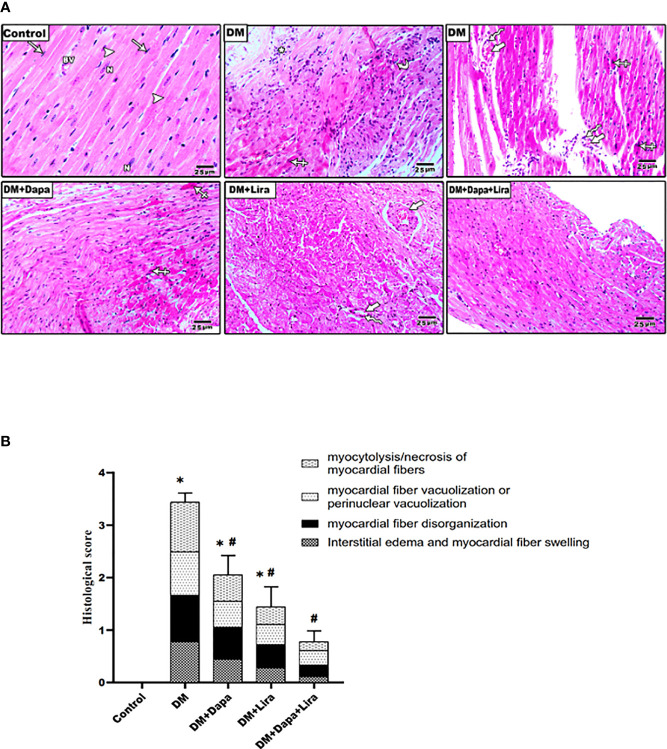
**(A)** Representative photomicrographs of H&E-stained cardiac sections from different experimental groups. Normal control rats showed normal histological structure of the cardiac muscle diabetic group demonstrating disarray of the cardiac myocytes with myocardial fiber disorganization (curved arrow), myocardial fiber necrosis with chronic inflammatory cells in the subpericardium (astrix), apoptotic myocyte with hypereosinophilic cytoplasm, pyknotic nuclei (crossed arrow), increased intermyocyte (thick arrows), and perivascular chronic inflammatory cells (zigzag arrows) marked interstitial edema. Treatment groups (DM+Dapa) and (DM+Lira) showed less structural injury. Combination therapy group (DM+Dapa+Lira) showed almost restoration of normal cardiac structure. ×400 bar 25. **(B)** A graph showing histological score for cardiomyopathy. Data are expressed as mean ± SEM (n = 6). *p < 0.05 versus control and ^#^ *p* < 0.05 versus diabetic group. Dapa, dapagliflozin; Lira, liraglutide.

As shown in **Figure 2A**, H&E-stained heart sections from the control group demonstrated the normal histological structure of the cardiac muscle fibers. Sections from diabetic rat hearts showed disarray of the cardiac myocytes with myocardial fiber disorganization, myocardial fiber necrosis, and mild chronic inflammatory cells in the subpericardium, apoptotic myocyte with hypereosinophilic cytoplasm, pyknotic nuclei and increased intermyocyte, interstitial edema, and perivascular chronic inflammatory cells. The degree of injury appears to be moderate in the DM+Dapa-treated group and mild in the DM+Lira-treated group. The normal structure in cardiac sections from the DM+Dapa+Lira group was almost restored. Additionally, these results were further ascertained by morphometric analysis shown in **Table 1** and **Figure 2B**.

**Table 1:** Histopathological scores for myocardial necrosis among different experimental groups.

	Myocardial fiber swelling and interstitial edema	Disorganization of myocardial fiber with or without fibroblastic proliferation	Perinuclear vacuolization or myocardial fiber vacuolization	Myocardial fibers myocytolysis/necrosis	Total severity score
**Control**	0.00 ± 0.00	0.00 ± 0.00	0.00 ± 0.00	0.00 ± 0.00	0.00 ± 0.00
**DM**	0.78 ± 0.14^*^	0.89 ± 0.08^*^	0.83 ± 0.09^*^	0.94 ± 0.06^*^	3.44 ± 0.17^*^
**DM+Dapa**	0.44 ± 0.13^*#^	0.66 ± 0.11^*#^	0.50 ± 0.12^*#^	0.50 ± 0.13^*#^	2.06 ± 0.37^*#^
**DM+Lira**	0.28 ± 0.10^*#^	0.45 ± 0.12^*#^	0.39 ± 0.12^*#^	0.33 ± 0.11^*#^	1.44 ± 0.38^*#^
**DM+Dapa+Lira**	0.06 ± 0.03^#^	0.23 ± 0.10^#^	0.28 ± 0.11^#^	0.17 ± 0.09^#^	0.78 ± 0.21^#^

Tissue pathological changes within each slide were inspected and scored in three high-power fields based on severity and extent of myocardial injury. A semiquantitative scoring from 0 to 3 or more was used, with no damage = 0 and severe damage = 3 or more. Values represent the average score for each animal. Results are considered significantly different when p < 0.05. Data are expressed as mean ± SEM (n = 6).

DM, diabetes mellitus; Dapa, dapagliflozin; Lira, liraglutide.

^*^p < 0.05 versus control.

^#^p < 0.05 versus diabetic group.

### Effects of Dapagliflozin–Liraglutide Treatment on Oxidative Stress Markers in Cardiac Tissue

**Figure 3:**
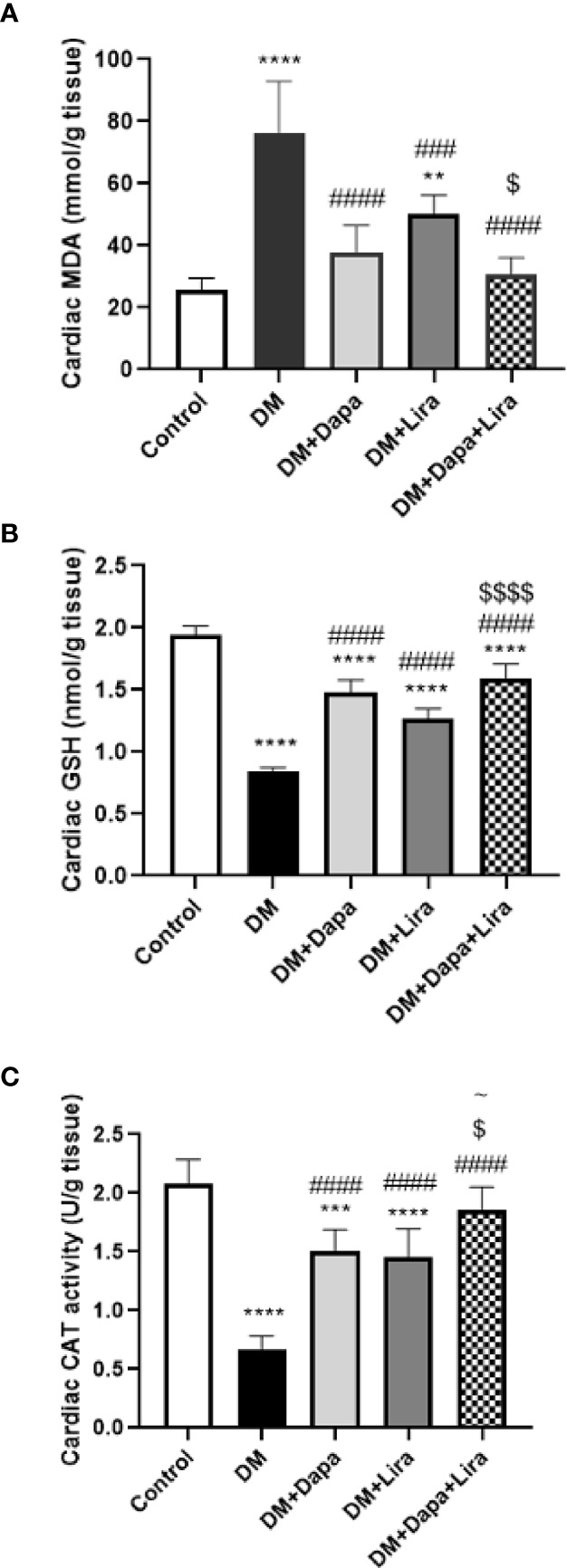
Effect of dapagliflozin (Dapa) and liraglutide (Lira) or their combination on oxidative stress biomarkers in rat heart tissues: **(A)** tissue malondialdehyde (MDA), **(B)** tissue reduced glutathione (GSH), and **(C)** tissue catalase activity (CAT). Data are represented as mean ± SE, n = 6. ** significance in comparison with control group at *p* < 0.01, *** at *p* < 0.001, **** at *p* < 0.0001, ### significance in comparison with diabetic group at *p* < 0.001, #### at *p* < 0.0001, ^~^ significance in comparison with DM+Dapa at *p* < 0.05, $ significance in comparison with DM+Lira group at *p* < 0.05, $$$$ at *p* < 0.0001.

Cardiac tissue levels of MDA demonstrated a marked increase (*p* < 0.0001) in the DM group in comparison with the normal control group. Meanwhile, a significant decrease in the treated groups (DM+Dapa and DM+Lira groups) (*p* < 0.0001) was observed as compared to that of the untreated DM group. Additionally, the combination treatment produced a significant decrease in MDA as compared to the DM+Lira group (*p* < 0.05). In contrast, the level of the antioxidant GSH and activity of antioxidant enzyme CAT were significantly decreased in cardiac tissues of the DM group (*p* < 0.0001) in comparison with the normal control group. However, a significant increase in the levels of these antioxidant moieties was observed in the treated groups (DM+Dapa and DM+Lira groups) when compared to the untreated DM group (*p* < 0.0001). Also, the combination therapy markedly increased tissue GSH (*p* < 0.0001) as compared to the DM+Lira group as well as tissue CAT (*p* < 0.05) compared to the DM+Dapa and DM+Lira groups (**Figures 3A–C**).

### Effect of Dapagliflozin–Liraglutide Treatment on mRNA Expression of TNF-α and Caspase-3 in Cardiac Tissue

**Figure 4:**
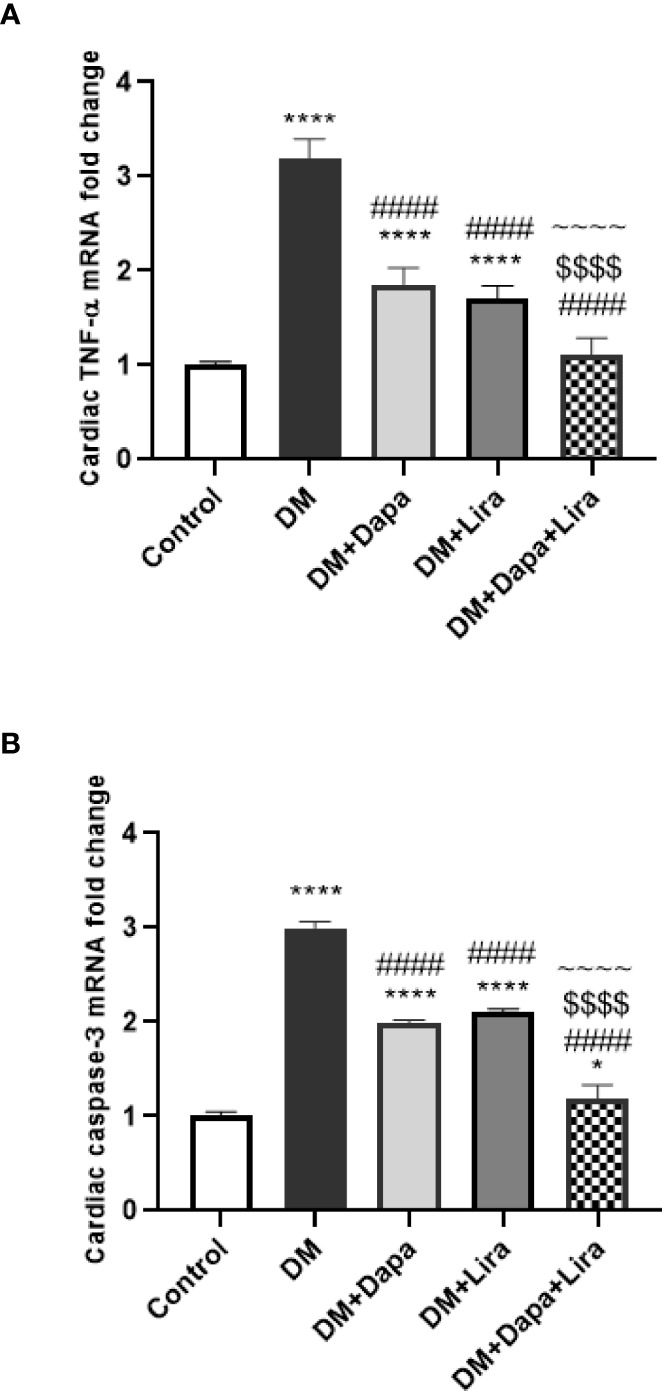
Effect of dapagliflozin (Dapa) and liraglutide (Lira) or their combination on mRNA expression of **(A)** tissue TNF-α and **(B)** tissue caspase-3. Data are represented as mean ± SE, n = 5. **** significance in comparison with control group at *p* < 0.0001, #### significance in comparison with diabetic group at *p* < 0.0001, ^~~~~^ significance in comparison with DM+Dapa at *p* < 0.0001, $$$$ significance in comparison with DM+Lira group at *p* < 0.0001.

TNF-α mRNA levels were significantly increased in the cardiac tissue of the DM group (*p* < 0.0001) compared to normal control. On the other hand, the pro-inflammatory cytokine mRNA levels were markedly decreased in the treated groups (DM+Dapa and DM+Lira groups, *p* < 0.0001) compared to the DM group. Additionally, the combination treatment resulted in a significant decrease in TNF-α mRNA level when compared with single treatment, *p* < 0.0001 (**Figure 4A**).

Regarding caspase-3, the mRNA levels of the apoptotic enzyme were significantly elevated in cardiac tissues of the DM group, *p* < 0.0001, in comparison with those of the normal control group. However, a marked decrease in its level was observed in groups treated with Dapa or Lira, *p* < 0.0001, as compared to the untreated DM group. Further, the combination therapy resulted in a more significant reduction in caspase-3 mRNA level compared to single treatment groups, *p* < 0.0001 (**Figure 4B**).

### Effect of Dapagliflozin–Liraglutide Treatment on Cardiac Tissue Inflammatory Cytokines IL-1β and IL-6

**Figure 5:**
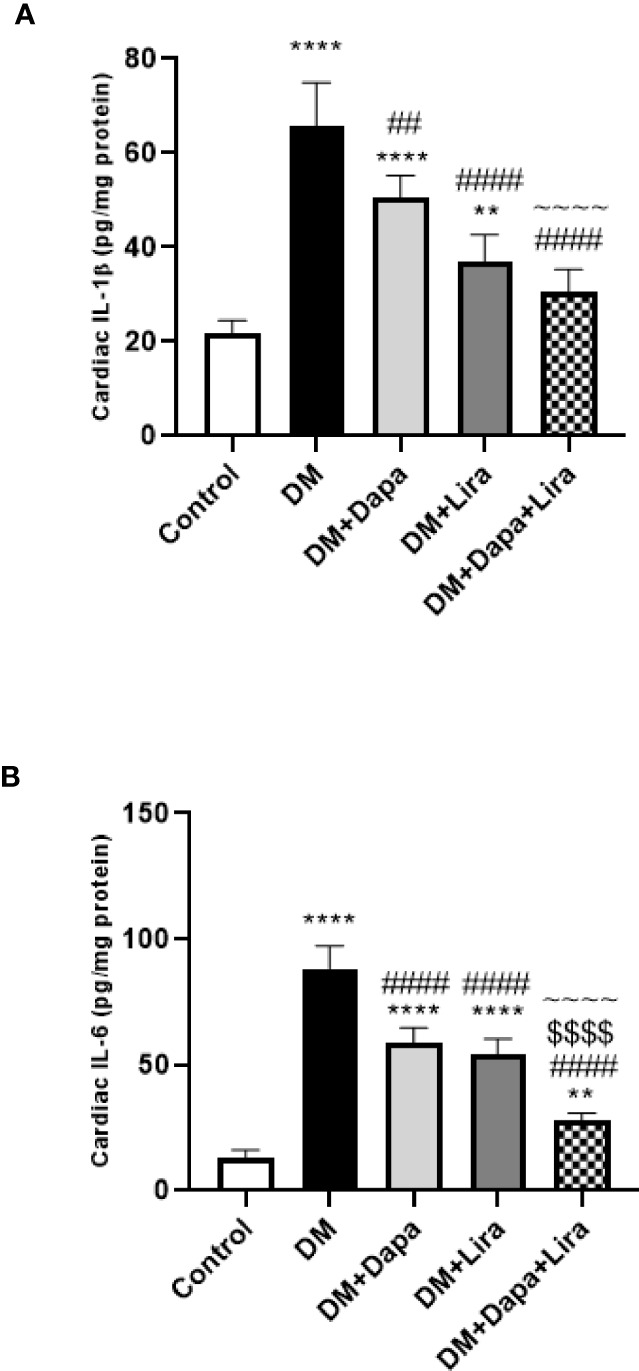
Effect of dapagliflozin (Dapa) and liraglutide (Lira) or their combination on protein levels of **(A)** tissue IL-1β and **(B)** tissue IL-6. Data are represented as mean ± SE, n = 5. ** significance in comparison with control group at *p* < 0.01, **** at *p* < 0.0001, ## significance in comparison with diabetic group at *p* < 0.01, #### at *p* < 0.0001, ^~~~~^ significance in comparison with DM+Dapa at *p* < 0.0001, $$$$ significance in comparison with DM+Lira group at *p* < 0.0001.

Cardiac tissue of the DM group demonstrated significantly increased levels of inflammatory cytokines IL-1β and IL-6 (*p* < 0.0001) as compared to normal control. However, the pro-inflammatory cytokine levels were significantly decreased in cardiac tissue of the DM+Dapa group (*p* < 0.01, *p* < 0.0001, respectively) and DM+Lira group (*p* < 0.0001) as compared to the untreated DM group. Also, the combination treatment group demonstrated a significant decrease in cardiac IL-1β levels (*p* < 0.0001) when compared with the DM+Dapa group and in cardiac IL-6 levels (*p* < 0.0001) when compared with treatment groups (DM+Dapa and DM+Lira groups) (**Figure 5**).

### Effect of Dapagliflozin–Liraglutide Treatment on Immunostaining of NF-κB/p56, TNF-α, and Cleaved Caspase-3 in Cardiac Tissue

**Figure 6:**
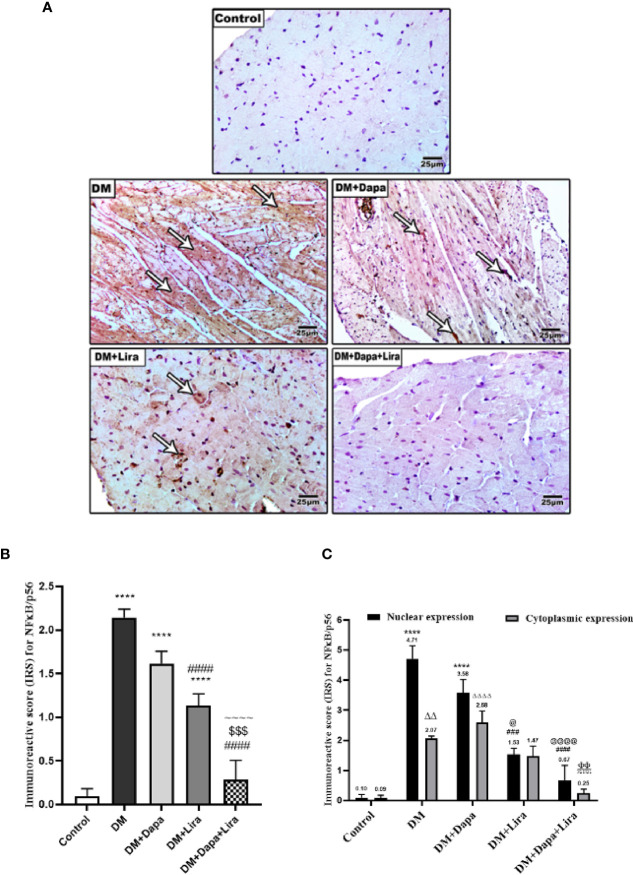
**(A)** Representative photomicrographs of NF-κB/p56 immuno-stained cardiac sections from different experimental groups, ×200 bar 50. **(B)** A graph showing immunoreactive score for NF-κB/p56 cellular expression. Data are represented as mean ± SE, n = 6. **** significance in comparison with control group at *p* < 0.0001, #### significance in comparison with diabetic group at *p* < 0.0001, ^~~~~^ significance in comparison with DM+Dapa at *p* < 0.0001, $$$ significance in comparison with DM+Lira group at *p* < 0.001. **(C)** A graph showing immunoreactive score for NF-κB/p56 differentially in the nucleus and cytoplasm. Data are expressed as mean ± SEM (*n* = 6). ^****^ *p* < 0.0001 versus control, ^####^ *p* < 0.0001, ^###^ *p* < 0.001 versus diabetic group and *p* < 0.0001, ^@^ *p* < 0.05 versus DM+Dapa for nuclear expression, *^ΔΔ^p* < 0.01, *^ΔΔΔΔ^p* < 0.0001 versus control, ^ΦΦ^ *p* < 0.01 versus diabetic group, ^πππ^*p* < 0.001 versus DM+Dapa for cytoplasmic expression. Dapa, dapagliflozin; Lira, liraglutide.

Results from IHC further reinforce RT-PCR findings. Indeed, immunostaining of cardiac tissues from different experimental groups demonstrated increased immunostaining of NF-κB/p56 in cardiac tissue of the diabetic group (*p* < 0.0001) when compared to normal control. The immunostaining was markedly decreased in treatment groups (DM+Dapa and DM+Lira groups, *p* < 0.0001) compared to the diabetic group. Additionally, the combination treatment resulted in a significant decrease in immunostaining of NF-κB/p56 in cardiac tissue compared with the DM+Dapa group (*p* < 0.0001) and DM+Lira group (*p* < 0.001) (**Figures 6A, B**). Further, the IRS score was used to compare the nuclear and cytoplasmic expression of NF-κB/p56 among different experimental groups. As shown in **Figure 6C**, the nuclear expression of NF-κB/p56 in the diabetic group was significantly different as compared to normal control (*p* < 0.0001). Treatment with Lira resulted in a significant reduction in NF-κB/p56 nuclear expression compared to the diabetic group (*p* < 0.001). Further, combination treatment resulted in a significant reduction in nuclear expression of NF-κB/p56 as compared to the DM+Dapa-treated group (*p* < 0.0001).

**Figure 7:**
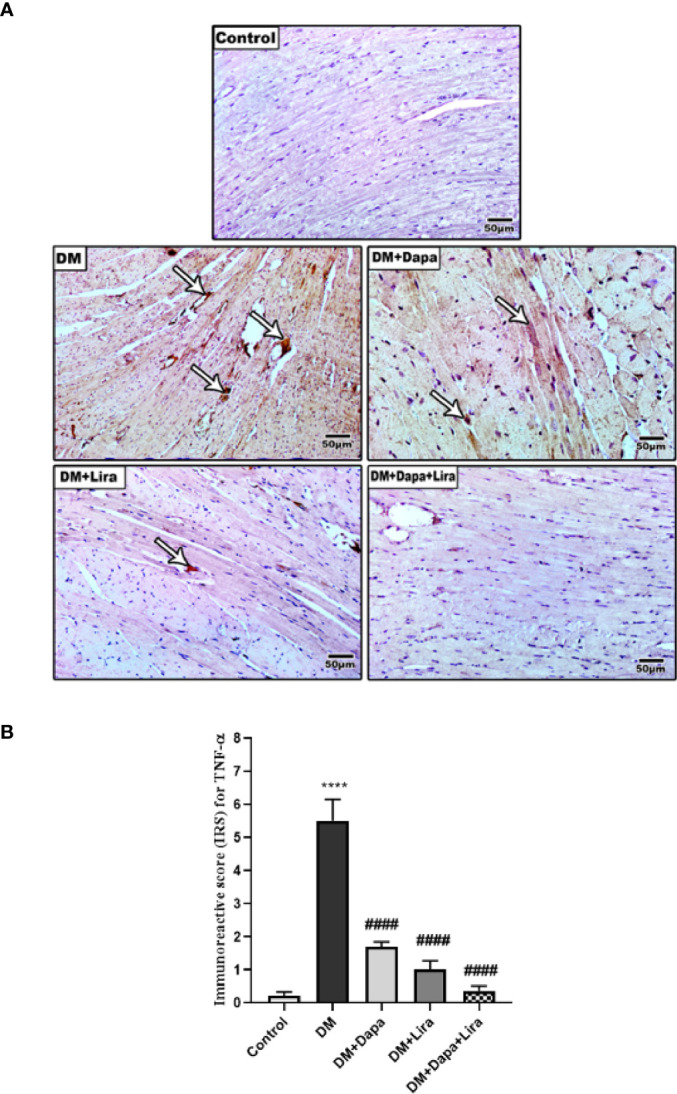
**(A)** Representative photomicrographs of TNF-α immuno-stained cardiac sections from different experimental groups, ×200 bar 50. **(B)** A graph showing immunoreactive score for TNF-α cellular expression. Data are represented as mean ± SE, n = 6. **** significance in comparison with control group at *p* < 0.0001, #### significance in comparison with diabetic group at *p* < 0.0001. Dapa, dapagliflozin; Lira, liraglutide.

**Figure 8:**
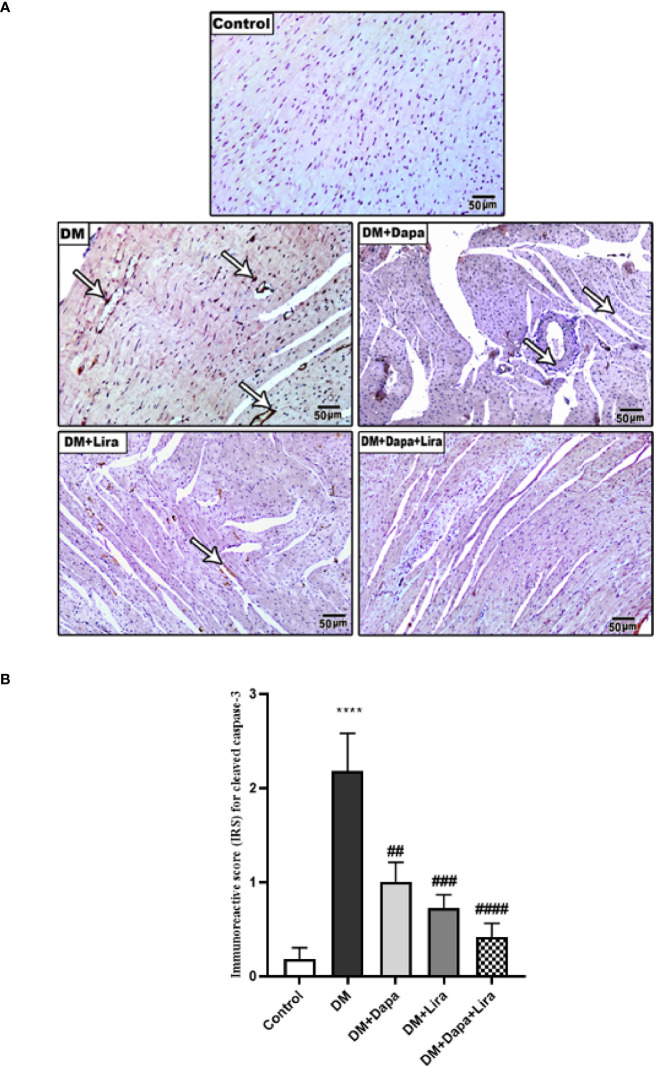
**(A)** Representative photomicrographs of cleaved caspase-3 immuno-stained cardiac sections from different experimental groups. ×200 bar 50. **(B)** A graph showing immunoreactive score for cleaved caspase-3 cellular expression. **** Significance in comparison with control group at *p* < 0.0001, #### significance in comparison with diabetic group at *p* < 0.0001, ^###^ significance in comparison with diabetic group at *p* < 0.001, ## significance in comparison with diabetic group at *p* < 0.01.

Immunostaining of TNF-α and cleaved caspase-3 was significantly increased in cardiac tissue of the diabetic group (*p* < 0.0001) compared to normal control. However, immunostaining of TNF-α and cleaved caspase-3 was significantly decreased in cardiac tissue of treatment groups (DM+Dapa and DM+Lira groups and combination treatment groups, *p* < 0.0001) compared to the diabetic group (**Figures 7**, **8**).

## Discussion

Despite using insulin as the mainstay for T1DM treatment, non-insulin adjunct therapy is a new trend. The purpose of this therapy is to slow down autoimmune processes, to regulate glucagon secretion, and to protect pancreatic β cells. Thereby, the use of this therapy has improved glycemic control, provided nephroprotection, and protected vascular endothelium in both clinical and experimental studies The present study demonstrated the protective efficacy of a selective SGLT2 inhibitor Dapa and a long-acting GLP-1 receptor agonist Lira and their combination therapy in DM-accompanied organ injury (24).

Oxidative stress plays a crucial role in the pathogenesis of DM and its induced complications. Disruption of the normal homeostasis of free radicals has been implicated in the impairment of pancreatic β-cell function (25). Further, oxidation of glucose and non-enzymatic glycation of proteins trigger free radical formation, which in turn causes damage to macromolecules, cellular machinery, and antioxidant enzymes (26). Indeed, several *in vivo* and *in vitro* experimental studies reported that diabetes-accompanied metabolic abnormalities cause mitochondrial superoxide overproduction. This event is considered a central and major mediator of diabetes tissue damage *via* activation of key pathogenic pathways involved in the pathogenesis of diabetic organ complications (27). Besides, oxidative stress inflammation is a common feature of DM. Both effectors reportedly share the same stimulus, reactive oxygen species (ROS). Of note, oxidative stress triggers inflammatory cascades, which in turn promote ROS production, creating a vicious cycle that increases the complexity of diabetes-associated multi-organ complications (28). Furthermore, DM-related oxidative stress and inflammation can trigger cellular apoptosis leading to cellular damage and organ failure (29). In agreement, we previously reported signs of oxidative stress, inflammation, and apoptosis in diabetic tissue using the same experimental model (30–33). Currently, our data were consistent as evident by an increased tissue biomarker of oxidative stress, MDA, and reduced cellular antioxidant defense, GSH and CAT. This was accompanied by increased tissue pro-inflammatory cytokine expression TNF-α as well as upregulated expression of the apoptotic enzyme, caspase-3.

In addition to its role as a crucial modulator of the inflammation process *via* regulation of the expression of hundreds of genes involved in cellular inflammatory events, NF-κB is also a redox-sensitive nuclear factor that subsequently modulates a large number of processes to maintain tissue homeostasis. Thereby, NF-κB possesses a strategic position at the crossroad between inflammation and oxidative stress, emphasizing its role as a potential target for the management of DM-accompanied organ injury (34). Herein, our data showed upregulated expression of NF-κB along with increased NF-κB/p56 immunostaining in rat diabetic cardiac tissues.

Accumulating evidence demonstrated therapeutic and protective efficacies of Dapa and Lira in T2DM-associated tissue injury. Chen et al. reported that Dapa administration protected against DM-induced oxidative stress in lens (35). Wei et al. demonstrated that Dapa improved pancreatic β-cell function in db/db mice (36). Tang et al. reported antioxidant and anti-inflammatory effects of Dapa with subsequent inhibition of glomerulosclerosis and liver fibrosis in db/db mice (37). In T2DM patients, Dapa administration resulted in beneficial outcomes pertaining to the microvascular sequelae including improvement of the renal resistive index, arterial stiffness, and systemic endothelial function (38, 39). With respect to Lira, it has been found to slow down memory function decline (40), improve cardiovascular and kidney outcomes, and reduce mortality in T2DM patients (41). Moreover, Lira treatment ameliorated the severity of T2DM complicated with non-alcoholic fatty liver disease (42). In experimental models of T2DM, Lira treatment protected against cognitive deficits (43) and exerted a renoprotective effect (44) *via* autophagy activation and endoplasmic reticulum stress attenuation (45). Moreover, Lira administration has modulated the gut microbiome and ameliorated fatty liver in db/db mice (46). Interestingly, a recent study using Dapa–Lira combined therapy showed beneficial metabolic and neuroprotective effects in diet-induced diabetic mice (17). Additionally, a clinical trial performed by Petrie et al. demonstrated that Dapa administration reduced cardiovascular morbidity and mortality in patients with heart failure independent of DM (47). Qin et al. reported protective effects of Dapa against the development of ventricular arrhythmia in pulmonary artery hypertension rats. The mechanism involves modulation of TLR4/NF-κB signaling pathway (48). Similarly, Lira has been reported to protect against myocardial pyroptosis in diabetic rats *via* activation of Sirt1/AMPK signaling pathways (49).

Given the aforementioned evidence and due to their multiple beneficial effects, Dapa and Lira seem to be a very attractive treatment option in T1DM. Indeed, using Dapa in combination with insulin and Lira resulted in a marked improvement in blood glucose and weight loss in T1DM patients (13). Moreover, Dapa treatment demonstrated an anti-atherogenic effect in T1DM mice (50). Interestingly, administration of Dapa or Lira to T1DM mice enhanced β-cell proliferation and decreased β-cell apoptosis (51). Similarly, our data revealed that administration of Dapa and/or Lira to diabetic animals attenuated accompanying cardiac tissue injury *via* modulating key elements of oxidative stress, inflammation, and apoptosis. Combination treatment was more effective as compared to sole treatment.

**Figure 9:**
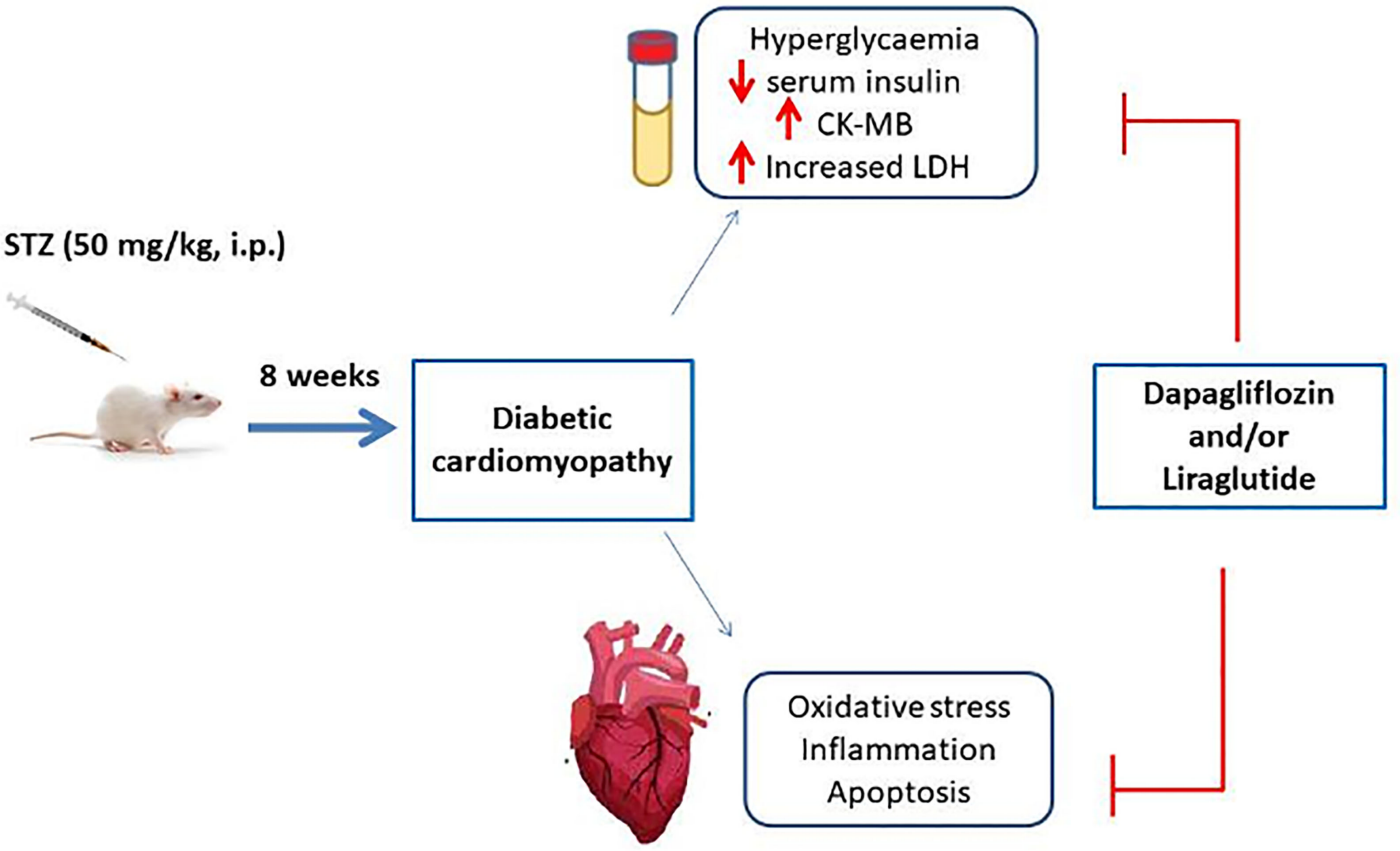
Graphical abstract.

In conclusion, the present study demonstrated beneficial protective effects of Dapa and/or Lira administration against T1DM-related cardiac injury. The mechanisms underlying these effects are attenuating oxidative stress, downregulated inflammation, and apoptosis. Both non-insulin pharmacological drugs could be used as adjunct therapy given their beneficial protective effects on body organs during the diabetic course (**Figure 9**).

## Data Availability Statement

Data are available upon request from the corresponding author.

## Ethics Statement

The animal study was reviewed and approved by the Research Ethics Committee, Faculty of Medicine, Mansoura University, Egypt.

## Author Contributions

Conceptualization, ME-Sha, ME-A, M-She; Funding acquisition, HE, M-She; Investigation, ME-Sha, ME-A, ME, HE, DE, ME-She, SA and NE; Methodology; ME-Sha, ME-A, ME, HE, DE, ME-She; Resources, ME-Sha, ME-A, ME, HE, DE, ME-She, SA and NE; Software, ME-Sha, ME-A, ME, HE, DDE, ME-She, SA and NE; Visualization, HE, ME-She, SA; Writing – original draft, ME-Sha, MA-A, DDE, ME-She, NE; Writing – review & editing, ME-Sha, ME-A, DE, ME-Sha, NE.

## Funding

The authors acknowledge the support provided by the Researchers Supporting program (TUMA, Project-2021-3), AlMaarefa University, Riyadh, Saudi Arabia. The present study was funded by Princess Nourah bint Abdulrahman University Researchers Supporting Project number (PNURSP2022R171), Princess Nourah bint Abdulrahman University, Riyadh, Saudi Arabia.

## Conflict of Interest

The authors declare that the research was conducted in the absence of any commercial or financial relationships that could be construed as a potential conflict of interest.

## Publisher’s Note

All claims expressed in this article are solely those of the authors and do not necessarily represent those of their affiliated organizations, or those of the publisher, the editors and the reviewers. Any product that may be evaluated in this article, or claim that may be made by its manufacturer, is not guaranteed or endorsed by the publisher.
